# Establishing a baseline of antibiotic use in Massachusetts jails reveals heterogeneity in quantity of prescriptions and duration of therapy

**DOI:** 10.1017/ash.2023.175

**Published:** 2023-06-21

**Authors:** Bart R. Szewczyk, Rebecca Tenner, Emily Grussing, Maureen Campion, Gabriela Andujar Vazquez, Tara Bylsma, Shira Doron, Alysse G. Wurcel

**Affiliations:** 1 Department of Public Health and Community Medicine, Tufts University School of Medicine, Boston, Massachusetts; 2 Tufts University, Medford, Massachusetts; 3 Tufts University School of Medicine, Boston, Massachusetts; 4 Division of Geographic Medicine and Infectious Disease, Department of Medicine, Tufts Medical Center, Boston, Massachusetts

## Abstract

Optimizing antibiotic prescribing is a crucial element of the fight against antibiotic resistance. Antibiotic prescribing patterns in jails have not been studied. We established a baseline of antibiotic prescribing between Massachusetts jails. We detected heterogeneity in quantity and duration of antibiotic prescriptions, revealing an opportunity for improved practice.

Antimicrobial resistance is a growing public health emergency that has sparked collaboration and innovation across disciplines. Efforts to improve antibiotic prescribing practices have focused on traditional medical settings, such as hospitals, with an increasing focus on emergency departments, ambulatory clinics, and skilled nursing facilities.^1^ However, antibiotic prescribing in correctional healthcare settings, including jails and prisons, has gone largely unstudied. Although core elements of antimicrobial stewardship have been recommended by the CDC that include application to low-resource settings, there are no guidelines for antimicrobial stewardship in correctional facilities. Approximately 2 million people are incarcerated each year in the United States, so investment in improving antimicrobial delivery to people who are incarcerated will benefit a sizeable portion of the country.^2^ We compared antibiotic use across Massachusetts jails as a first step to improve antibiotic prescribing practices.

## Methods

### Data set

We collected deidentified antibiotic prescription data (drug name, dose frequency, number of doses, order start and end date) for all 12 county jails in Massachusetts for the 2021 calendar year. One jail was excluded due to its relatively small size and census. The data set was analyzed across 4 categories of oral and intramuscular antibiotic prescriptions: (1) anti-MRSA agents (clindamycin, doxycycline hyclate, doxycycline monohydrate, linezolid, minocycline, tetracycline HCl, sulfamethoxazole-trimethoprim), (2) fluoroquinolones (ciprofloxacin and levofloxacin), (3) other antibiotics (amoxicillin, azithromycin, cefadroxil, cefuroxime axetil, cefpodoxime proxetil, ceftriaxone, cephalexin, clarithromycin, erythromycin, and penicillin), and (4) all.

### Analysis

We chose 2 metrics for comparison of antibiotic use between jails: antibiotic days per patient and an estimated defined daily dose (DDD) per 1,000 inhabitants. Antibiotic days per patient was used to model the most prescribed duration of therapy. The DDD methodology is recommended by the WHO and the CDC for monitoring medication use.^3^ Due to the limited availability of jail census data, we estimated each jail’s population by converting the average daily population (ADP) to an annual population (Supplementary Fig. 2). The ADP was converted to an average weekly population, then multiplied by the average weekly turnover rate for a jail of that size to account for jail flux, and finally multiplied by 52 weeks.^4,5^

## Results

We detected heterogeneity in antibiotic prescribing practices across the Massachusetts state jail system. Our analysis revealed differences in the quantity of prescriptions and duration of therapy.

### Duration of therapy

The most frequently prescribed duration of therapy with antibiotics varied between jails. For fluoroquinolones, jail 2 had the shortest duration of therapy at 5 days, whereas jail 11 had the longest duration of therapy at 30 days (Supplementary Fig. 1). The duration of therapy with anti-MRSA drugs ranged from 7 days in some jail to 10 days in others. Duration of therapy for “other” and “all” antibiotics ranged from 7 to 14 days.

### Quantity of prescriptions

**Figure 1.**
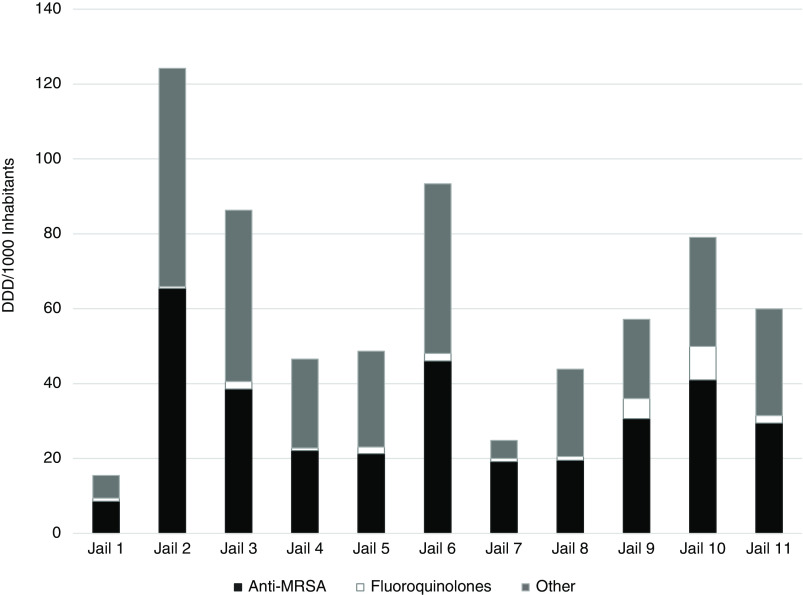
The defined daily dose (DDD) of 19 antibiotics prescribed across 11 Massachusetts jails in 3 categories: anti-MRSA agents (clindamycin, doxycycline hyclate, doxycycline monohydrate, linezolid, minocycline, tetracycline HCl, sulfamethoxazole-trimethoprim), fluoroquinolones (ciprofloxacin, levofloxacin), and other (amoxicillin, azithromycin, cefadroxil, cefuroxime axetil, cefpodoxime proxetil, ceftriaxone, cephalexin, clarithromycin, erythromycin, and penicillin).

The estimated DDD per 1,000 inhabitants for “all” antibiotics ranged from 15.5 to 124.2 (Fig. 1). Jail 1 had the lowest estimated DDD per 1,000 inhabitants of 15.5. The equivalent of 1.5% (15.5 per 1,000) of the population in jail 1 received an antibiotic that year. Jail 2 had the highest estimated DDD per 1,000 inhabitants of 124.2; that is, the equivalent of 12.4% of the population of jail 2 received an antibiotic that year.

## Discussion

Variability in prescribing practices reflects an opportunity to optimize antibiotic prescribing.^6^ Although further investigation into the indication for therapy is needed, evaluating duration of therapy and selection of antibiotics can help to identify specific areas for detailed review. For example, anti-MRSA antibiotic use contributed to 50% or greater of the antimicrobial DDD per jail (Fig. 1), with 10 days of therapy as the common mode for many jails (Supplementary Fig. 1). For uncomplicated skin and soft-tissue infections, a common indication for oral anti-MRSA treatment, shorter courses of therapy (5–7 days) are recommended.^7^ Therefore, there may be an opportunity to educate clinicians on the selection of agents within this setting.

Contrary to jails, the federal prison system has developed strategies aimed at improving antibiotic prescribing, including implementation of antimicrobial stewardship programs (ASPs).^8^ Although ASPs may exist at jails or state prisons, we could find no publicly available data about the process or outcomes. Better understanding antibiotic prescribing practices in the carceral healthcare setting comes with an additional urgency owing to the significant health equity and public health concerns associated with the incarcerated population. The carceral system is the primary health provider to a large portion of the United States, with disproportionate incarceration of poor, minoritized, and marginalized communities.^9^ Carceral facilities in the United States have high rates of multidrug-resistant infections, and prudent antibiotic use in these settings is highly relevant.^10^

This study had several limitations. Jail healthcare delivery models vary by county in Massachusetts (ie, direct oversight vs medical vendor oversight), and this was not considered in our analysis. Because our data unit was deidentified prescriptions, we could not ascertain nor control for how pre-existing conditions affected antibiotic utilization nor evaluate the appropriateness of antibiotic prescriptions.

Despite these limitations, our research is novel as the first publication evaluating variability in prescribing practices among jails within the same state system. Having established this baseline of antibiotic use, our next steps will include analyzing disaggregated data to identify underlying trends and appropriateness of prescriptions. Our goal is to work with key stakeholders in jail and prison healthcare to promote integration of antimicrobial stewardship practices, decrease exposure to unnecessary antibiotics, and decrease the development of resistant bacteria.

## Supplementary material

For supplementary material accompanying this paper visit http://doi.org/10.1017/ash.2023.175. click here to view supplementary material
